# Supplementary material for: Study on Metronidazole Acid-Base Behavior and Speciation with Ca2+ for Potential Applications in Natural Waters
Source: Molecules. 2022 Aug 24;27(17):5394. doi: 10.3390/molecules27175394 (PMC9457533; doi:10.3390/molecules27175394)
Supplement: Supplementary file 1 [file molecules-27-05394-s001.zip › molecules-1855138-supplementary.pdf]

Supplementary Material

Table S1. Hydrolysis Constants of Ca^{2+} at different temperatures and ionic strength values.

**Species**	***t*** **/°C**	***I*** **/ mol L**$^{-1}$	**log**β[1, 2]
$CaOH^+$	15	0.15	-13.14
	25	0.15	-12.87
		0.5	-12.88
		1	-12.81
	37	0.15	-12.56

[1] Refer to reaction: $Ca^{2+} + H_2O = CaOH^+ + H^+$;.

[2] Crea, F.; De Stefano, C., Milea, D., Pettignano, A., Sammartano, S. (2015). SALMO and S3M: A Saliva Model and a Single Saliva Salt Model for Equilibrium Studies, Bioinorg. Chem. Appl. 2015.

Table S2. Calculated chemical shift (in ppm) of each nucleus of *MNZ*(L) species at t = 25 °C and I = 0.15 mol L^{-1} in NaCl.

	**L**	**LH**	**LH_2**
δ_{CH}	8.02(2)	8.01(2)	8.48(2)
δ_{CH3}	2.45(2)	2.45(2)	2.74(2)
δ_{CH2-1}	3.81(6)	3.87(6)	3.96(6)
δ_{CH2-2}	4.45(1)	4.45(1)	4.66(3)

Table S3. Formation constants of Cu^{2+}- and Zn^{2+}-*MNZ*(L) species at *t* = 25 °C and *I* = 0.7 mol L^{-1}.

logβ [1,2]				
**ZnL**	**ZnLH**	**ZnLOH**	**CuL**	**CuL_2**
7.00(5)	14.10(2)	-0.14(4)	9.0(4)	17.9(1)

[1] Refer to the reactions: M + qL + H = MLqH; MOH + L = MLOH; [2] unpublished data from this laboratory.

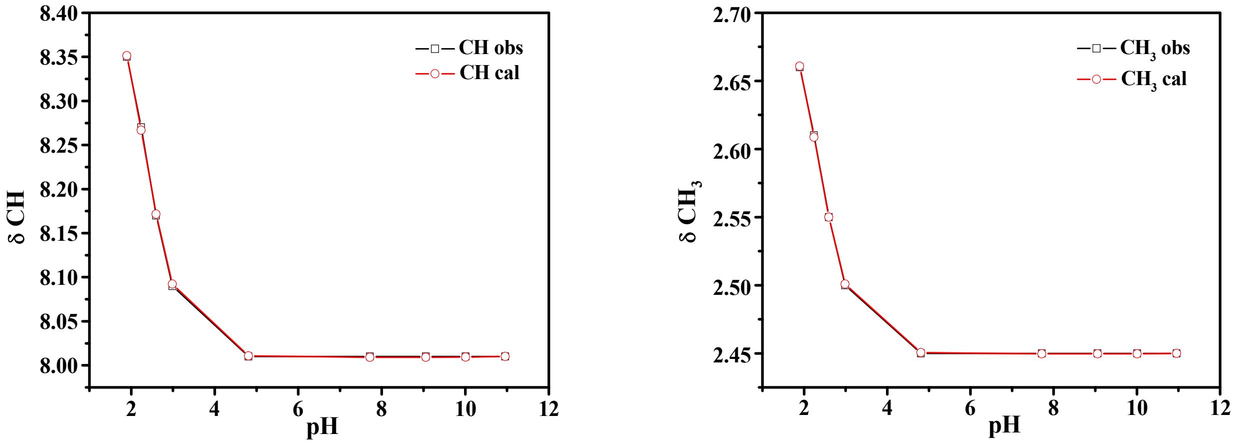

Figure S1. Comparison between experimental (□) and calculated (o) chemical shift values (in ppm) *vs*. pH of CH_3 and CH nuclei.

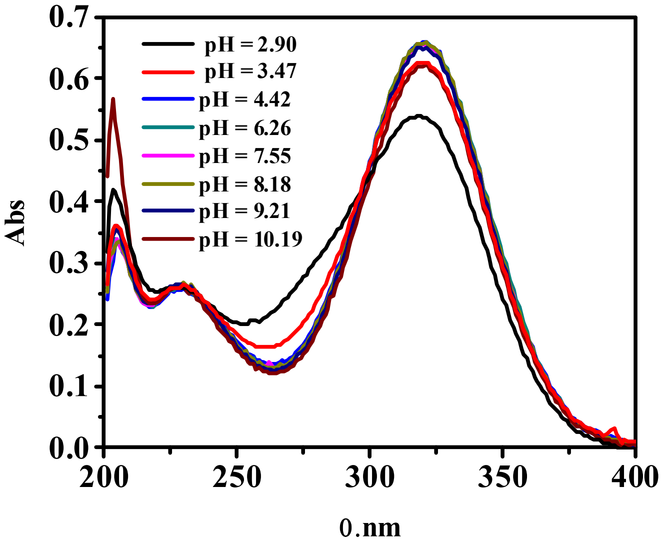

Figure S2. UV spectra at *t* =25 °C and *I* = 0.15 mol L^{-1} of Ca^{2+}-*MNZ*(L) solutions at C_L = 0.075 mmol L^{-1}, C_{Ca} = 0.0375 mmol L^{-1}.

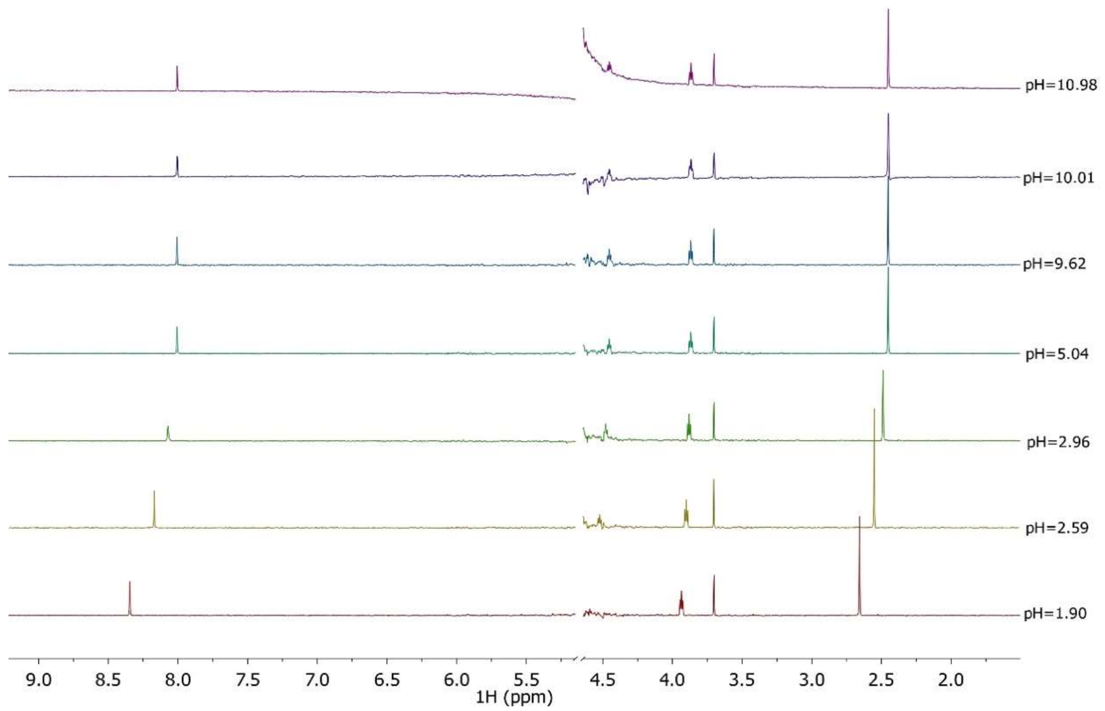

Figure S3. ^{1}H NMR spectra on solutions containing Ca^{2+}-*MNZ*(L) at C_L = 5 mmol L^{-1}, C_{Ca} = 6 mmol L^{-1}, *t* = 25 °C, *I* = 0.15 mol L^{-1} in NaCl, 1.90 ≤ pH ≤ 10.98.
